# Inhibition of Cdk5 increases osteoblast differentiation and bone mass and improves fracture healing

**DOI:** 10.1038/s41413-022-00195-z

**Published:** 2022-04-06

**Authors:** Mubashir Ahmad, Benjamin Thilo Krüger, Torsten Kroll, Sabine Vettorazzi, Ann-Kristin Dorn, Florian Mengele, Sooyeon Lee, Sayantan Nandi, Dilay Yilmaz, Miriam Stolz, Naveen Kumar Tangudu, David Carro Vázquez, Johanna Pachmayr, Ion Cristian Cirstea, Maja Vujic Spasic, Aspasia Ploubidou, Anita Ignatius, Jan Tuckermann

**Affiliations:** 1 Institute of Comparative Molecular Endocrinology (CME), Ulm University, Helmholtzstrasse 8/1, 89081 Ulm, Germany; 2 Institute of Orthopedic Research and Biomechanics, Ulm University, Helmholtzstrasse 14, 89081 Ulm, Germany; 3 Leibniz Institute on Aging – Fritz Lipmann Institute (FLI), Beutenbergstrasse 11, D-07745 Jena, Germany; 4 Praxisklinik für Orthopädie, Unfall- und Neurochirurgie Prof. Bischoff/Dr. Spies/Dr. Mengele, 89231 Neu-Ulm, Germany; 5 Paracelsus Medizinische Privatuniverstät, Institute of Pharmacy, Strubergasse 21, 5020 Salzburg, Austria; 6 Department of Endocrinology, Ludwig Maximilians University Munich, Munich, 80336 Germany; 7 Present Address: Institute of Orthopedic Research and Biomechanics, Ulm University, Helmholtzstrasse 14, 89081 Ulm, Germany; 8 Present Address: UPMC Hillman Cancer Center, Department of Pharmacology and Chemical Biology, University of Pittsburgh, 5115 Center Avenue, 15232 Pittsburgh, PA USA; 9 Present Address: TAmiRNA GmbH, Leberstrasse 20, 1110 Vienna, Austria

**Keywords:** Metabolic bone disease, Bone

## Abstract

Identification of regulators of osteoblastogenesis that can be pharmacologically targeted is a major goal in combating osteoporosis, a common disease of the elderly population. Here, unbiased kinome RNAi screening in primary murine osteoblasts identified cyclin-dependent kinase 5 (Cdk5) as a suppressor of osteoblast differentiation in both murine and human preosteoblastic cells. Cdk5 knockdown by siRNA, genetic deletion using the Cre-loxP system, or inhibition with the small molecule roscovitine enhanced osteoblastogenesis in vitro. Roscovitine treatment significantly enhanced bone mass by increasing osteoblastogenesis and improved fracture healing in mice. Mechanistically, downregulation of *Cdk5* expression increased Erk phosphorylation, resulting in enhanced osteoblast-specific gene expression. Notably, simultaneous *Cdk5* and *Erk* depletion abrogated the osteoblastogenesis conferred by *Cdk5* depletion alone, suggesting that Cdk5 regulates osteoblast differentiation through MAPK pathway modulation. We conclude that Cdk5 is a potential therapeutic target to treat osteoporosis and improve fracture healing.

## Introduction

Osteoporosis, which results in an increased fracture risk and impaired fracture healing, is a major burden of a large proportion of the aging population. Osteoblasts are bone-forming cells that differentiate from mesenchymal progenitors^1^. The major osteoblast function is the synthesis of extracellular matrix and support of mineralization^2^. This function, in concert with the activity of bone-resorbing osteoclasts, is crucial for bone remodeling and bone mass maintenance. Dysregulation of bone formation and resorption leads to several bone disorders, including osteoporosis^3^, which is characterized by low bone mass and altered bone tissue microarchitecture, which together contribute to increased bone fragility and fractures^4^.

Antiresorptive medical treatments are common but do not improve age-related loss of bone formation, which impacts bone quality. Signaling pathways known to induce osteoblastogenesis are triggered by bone morphogenetic proteins, modulators of Wnt signaling, and parathyroid hormone (PTH) peptide application^5^. Both intermittent low-dose PTH treatment and antibody treatment against sclerostin to induce Wnt signaling enhance osteoblast activity^6,7^; however, they are prohibitively expensive compared to small molecule-based pharmaceuticals.

Most of the known osteoblast regulators have been identified via single-gene mutations in humans and mice^8–10^. To discover novel regulators and their associated pathways, researchers use reverse genetics, which forms a link that connects a gene product to its cellular functional processes. We previously developed a cell-based RNAi high-content screening method to identify novel regulators of osteoblast differentiation using primary murine osteoblasts^11^. We employed this method to screen an RNAi library of murine kinases, given that kinases are the most productive class of established drug targets; there are numerous clinically approved kinase inhibitors, and more are undergoing screening in ongoing clinical trials^12^.

Using this methodology, we identified cyclin-dependent kinase 5 (Cdk5) as a previously unknown suppressor of osteoblastogenesis. Cdk5 belongs to the proline-directed serine/threonine cyclin-dependent kinase (Cdk) family^13,14^. Cdk5, in contrast to other Cdks, does not normally participate in cell cycle control but can aberrantly modulate various cell cycle components upon its deregulation^15^. The two related proteins Cdk5r1 (p35) and Cdk5r2 (p39), which are highly expressed in postmitotic neurons, directly bind to Cdk5 for its activation^16,17^. Cdk5 knockout mice are embryonically lethal, displaying defects in cortical layering, suggesting its essential role in brain development^18^. However, the role of Cdk5 in bone homeostasis is unknown.

## Results

### RNAi screening identifies Cdk5 as a new suppressor of osteoblast differentiation and mineralization

**Fig. 1:**
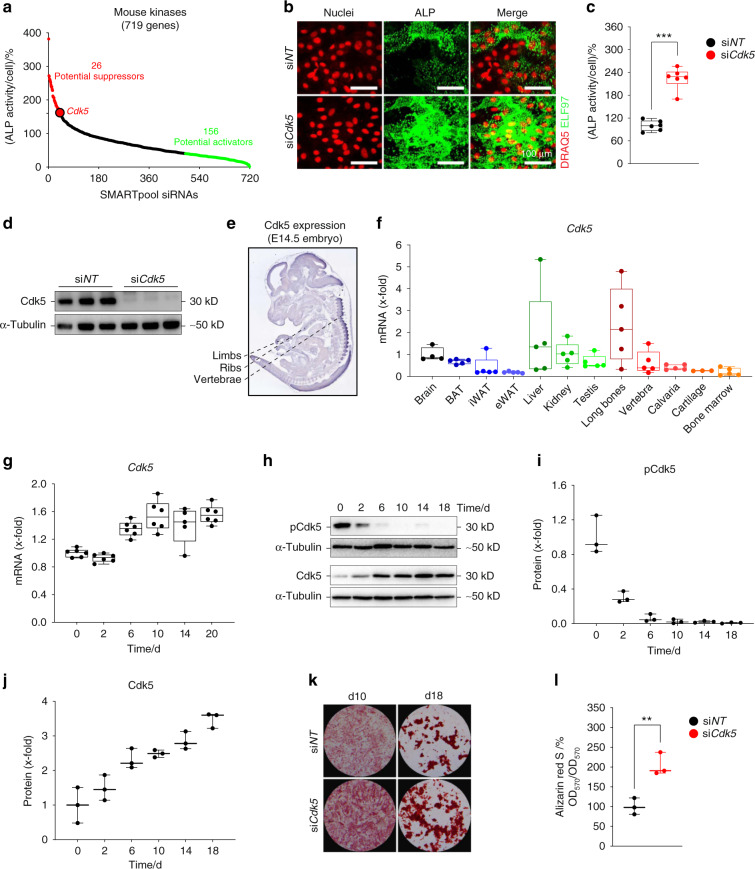
Identification of Cdk5 as a suppressor of osteoblast differentiation and mineralization. **a** Rank plot of the percentage of cellular ALP activity in primary osteoblasts after individual siRNA knockdown of 719 kinases (red dots represent siRNAs increasing ALP ≥ 160%, green dots represent siRNAs decreasing ALP ≤ 40%) **b** Representative microscopic images of primary murine calvarial osteoblasts upon *Cdk5* siRNA knockdown showing nuclear and ALP staining with DRAQ5 (red) and ELF 97 (green), respectively (scale bar: 100 µm). **c** Quantification of the percentage of cellular ALP activity upon *Cdk5* siRNA knockdown in primary murine calvarial osteoblasts (*n* = 6). **d** Western blot showing Cdk5 protein expression after 8 days of siRNA knockdown in primary murine calvarial osteoblasts (*n* = 3). **e** *Cdk5* expression in developing mice at embryonic day 14.5 (E14.5) by in situ hybridization using the online transcriptome atlas Eurexpress^20,21^. **f** qPCR analysis of *Cdk5* mRNA levels in the brain, brown adipose tissue (BAT), inguinal white adipose tissue (iWAT), epididymal white adipose tissue (eWAT), liver, kidney, testis, long bones, vertebra, calvaria, cartilage, and bone marrow (*n* = 3–5). **g** qPCR analysis of the *Cdk5* mRNA level during osteoblast differentiation in primary murine calvarial osteoblasts (*n* = 5–6). **h** Cdk5 phosphorylation (Cdk5^Tyr15^) and total protein levels by western blotting and, **i** and **j**, quantification during osteoblast differentiation in primary murine calvarial osteoblasts (*n* = 3). **k** Conventional ALP and Alizarin Red S staining (*n* = 3) from the primary murine calvarial osteoblasts transfected with non-targeting siRNA (si*NT*) or *Cdk5*-specific siRNA (si*Cdk5*) at 12 and 20 days post-transfection, respectively. **l** Quantification of Alizarin Red S by the acetic acid extraction method from the primary murine calvarial osteoblasts transfected with si*NT* or si*Cdk5* at 20 days post-transfection. Data are represented as box-and-whisker plots with min. to max. as well as with superimposition of all the data points. Statistical differences between groups were determined by unpaired homoscedastic two-tailed Student’s *t* test. *P < 0.05, **P < 0.01, ***P < 0.001

To identify novel genes crucial for osteoblast differentiation, we performed an unbiased RNAi screen, targeting 719 mouse kinases in primary murine calvarial osteoblasts (Fig. 1a). As murine osteoprogenitors differentiate into osteoblasts, alkaline phosphatase (ALP) activity is increased in an early maturation step^11^. Therefore, the quantification of ALP activity by an established fluorescence-based method^11^ was applied to determine osteoblast differentiation. ALP levels of small interfering RNA (siRNA)-treated osteoprogenitor cells were evaluated after 8 days of culture and subsequently normalized to the cell number to obtain ALP levels per cell (Fig. 1a). On the basis of previously described criteria for hit selection^11^, we identified 26 potential suppressors and 156 activators of osteoblast differentiation (Fig. 1a, Table S1). Among the suppressors that enhanced ALP activity, we identified *Cdk5* (Fig. 1a), a regulator of osteoblast differentiation that has not been intensively investigated. We confirmed in validation experiments that *Cdk5*-specific siRNA (si*Cdk5*) enhanced the cellular ALP levels (Fig. 1b and c) with efficient Cdk5 deletion at the protein level (Fig. 1d). To exclude the possibility that any observed enhanced ALP activity was due to off-target effects of other Cdks, we evaluated the impact of *Cdk1*, *Cdk4*, and *Cdk6* on osteoblast differentiation from our siRNA screen. As expected, the siRNAs against *Cdk1* and *Cdk6* did not induce any changes in ALP activity, whereas the siRNA against *Cdk4* resulted in decreased osteoblast differentiation, which was in contrast to the *Cdk5* siRNA results, suggesting that *Cdk4* might support osteoblast differentiation (Fig. S1a–c).

Because Cdk5 is believed to function mainly in the brain^19^, we investigated whether *Cdk5* mRNA is also expressed in bone. Indeed, the Eurexpress atlas^20^ reports *Cdk5* mRNA expression by in situ hybridization at sites of bone development at embryonic day 14.5 (E14.5)^21^. The embryos expressed *Cdk5* in the vertebrae and limbs and partially in the ribs (Fig. 1e)^20,21^. Analysis of *Cdk5* mRNA expression in adult murine tissues showed higher levels in long bones and liver than in the brain (Fig. 1f). Moderate expression was also observed in brown adipose tissue and kidney and to a lesser extent in epididymal white adipose tissue, cartilage, and bone marrow (Fig. 1f). Furthermore, mRNA expression of *Cdk5*, together with its activators *Cdk5r1* (*p35*) and *Cdk5r2* (*p39*), was found in primary osteoblasts, with increased *Cdk5* and *Cdk5r1* expression during osteoblast differentiation (Fig. 1g; Fig. S2a and S2b). Interestingly, Cdk5 phosphorylation (Cdk5^Tyr15^) in primary murine calvarial osteoblasts decreased during osteoblast differentiation (Fig. 1h and i), whereas similar to *Cdk5* mRNA expression, the total Cdk5 protein increased (Fig. 1h and j). In addition to the increased cellular ALP activity (Fig. 1b and c), si*Cdk5* increased conventional ALP staining (Fig. 1k) and bone nodule formation compared with the non-targeting siRNA (si*NT*) control (Fig. 1k and l).

**Fig. 2:**
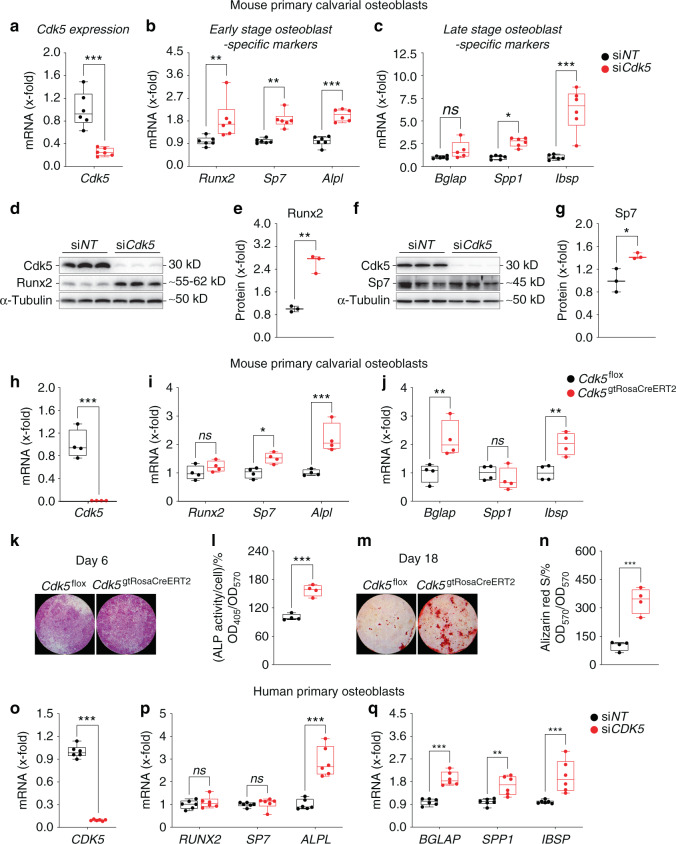
Cdk5 silencing or genetic deletion increases osteoblast-specific marker gene expression in primary murine and human osteoblasts. **a** *Cdk5* mRNA expression in primary murine calvarial osteoblasts after 8 days of siRNA transfection was quantified by qPCR (*n* = 6). **b** and **c** qPCR analysis of early-stage (*Runx2, Sp7, Alpl*) and late-stage (*Bglap, Ibsp, Spp1*) osteoblast-specific marker gene expression in the primary murine calvarial osteoblasts transfected with non-targeting siRNA (si*NT*) control or *Cdk5*-specific siRNA (si*Cdk5*) at 8 and 16 days post-transfection, respectively (*n* = 6). **d** and **e** Runx2 protein levels by western blotting and quantification, respectively, in the primary murine calvarial osteoblasts transfected with si*NT* or si*Cdk5* at 8 days post-transfection (*n* = 3). **f** and **g** Sp7 protein levels by western blotting and quantification, respectively, in the primary murine calvarial osteoblasts transfected with si*NT* or si*Cdk5* at 8 days post-transfection (*n* = 3). **h** *Cdk5* mRNA expression in the primary murine calvarial osteoblasts isolated from *Cdk5*^flox^ and *Cdk5*^gtRosaCreERT2^ pups after 8 days of seeding, quantified by qPCR (*n* = 4). **i** and **j** qPCR analysis of early-stage (*Runx2, Sp7, Alpl*) and late-stage (*Bglap, Ibsp, Spp1*) osteoblast-specific marker gene expression from the *Cdk5*^flox^ and *Cdk5*^gtRosaCreERT2^ primary murine calvarial osteoblasts after 8 and 16 days of culture, respectively (*n* = 4). **k** and **l** Qualitative and quantitative ALP staining, respectively, of the primary murine calvarial osteoblasts isolated from *Cdk5*^flox^ and *Cdk5*^gtRosaCreERT2^ pups after 8 days of culture (*n* = 4). **m** and **n** Qualitative and quantitative Alizarin Red S staining, respectively, from the primary murine calvarial osteoblasts isolated from *Cdk5*^flox^ and *Cdk5*^gtRosaCreERT2^ pups after 20 days of culture (*n* = 4). **o** *CDK5* mRNA expression in human primary osteoblasts at 12 days post-siRNA transfection quantified by qPCR (*n* = 6). **p** and **q** qPCR analysis of early-stage (*RUNX2, SP7, ALPL*) and late-stage (*BGLAP, IBSP, SPP1*) osteoblast-specific marker gene expression in the human primary osteoblasts transfected with si*NT* control or si*CDK5* at 12 days post-transfection, respectively (*n* = 6). Data are represented as box-and-whisker plots with min. to max. as well as with superimposition of all the data points. Statistical differences between two groups were determined by unpaired homoscedastic two-tailed Student’s *t* test and two-way ANOVA with Sidak’s multiple comparisons test. *P < 0.05, **P < 0.01, ***P < 0.001

Subsequently, we investigated the effects of *Cdk5* siRNA-mediated knockdown on osteoblast marker gene expression. Compared with that of the si*NT* control, the introduction of si*Cdk5* into primary murine calvarial osteoblast cultures resulted in a reduction in *Cdk5* mRNA expression (Fig. 2a), consistent with the protein reduction (Fig. 1d), without affecting the expression of other murine *Cdks* (Fig. S3a). This decreased *Cdk5* expression elevated the expression of early- (*Runx2, Sp7, Alpl*) and late-stage (*Spp1, Ibsp*) osteoblast-specific marker genes (Fig. 2b and c). We further confirmed these findings by introducing si*Cdk5* into primary murine calvarial osteoblast cultures, which elevated the Runx2 and Sp7 protein levels compared with those of the si*NT* control (Fig. 2d–g).

Subsequently, we determined whether *Cdk5* deletion by the *Cre-loxP* system in osteoblasts also improves osteoblast differentiation. Therefore, primary calvarial osteoblasts were isolated from *Cdk5*^flox^ and *Cdk5*^gtRosaCreERT2^ pups. Following 4-hydroxytamoxifen (4-OHT) treatment, the *Cdk5*-depleted (*Cdk5*^gtRosaCreERT2^) cells (Fig. 2h) exhibited increased expression of early- (*Sp7, Alpl*) and late-stage (*Bglap, Ibsp*) osteoblast-specific marker genes compared with the *Cdk5*^flox^ control cells (Fig. 2i and j). Moreover, qualitative and quantitative ALP staining and Alizarin Red S staining were significantly increased in the *Cdk5*^gtRosaCreERT2^ cells compared with the *Cdk5*^flox^ control cells (Fig. 2k–n).

To additionally test the effect of *CDK5* knockdown in a human system, we isolated primary osteoblasts from the medial head of the first metatarsal bone of a patient who had undergone surgery. si*CDK5*-mediated knockdown resulted in significantly reduced *CDK5* expression (Fig. 2o), without substantially altering the expression of other *CDKs* (Fig. S3b) compared with the si*NT* control. Consistent with our data from murine osteoblasts, the reduced *CDK5* expression in human osteoblasts subsequently increased early- (*ALPL*) and late-stage (*BGLAP, SPP1, IBSP*) osteoblast-specific marker gene expression (Fig. 2p and q).

These experiments demonstrated that *Cdk5* is expressed in osteoblasts and that its depletion augments in vitro osteoblast differentiation and mineralization from both mouse and human osteoprogenitor precursors.

### Cdk5 inhibition with roscovitine increases in vitro osteoblast differentiation and bone mass in vivo

**Fig. 3:**
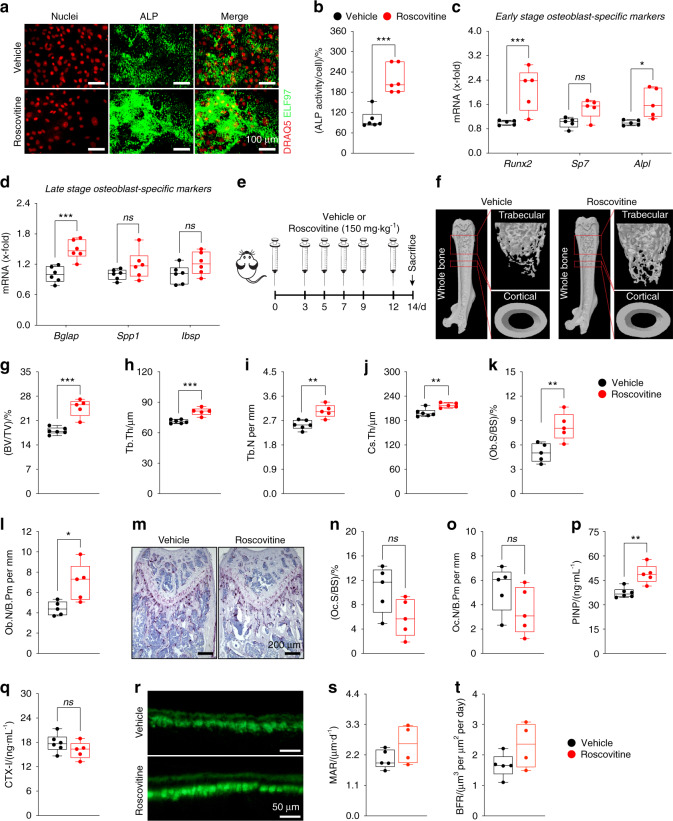
Roscovitine inhibition of Cdk5 augments in vitro osteoblast differentiation and enhances bone mass in vivo by increasing osteoblastogenesis. **a** Representative microscopic images showing nuclear and ALP staining in primary murine calvarial osteoblasts with DRAQ5 (red) and ELF97 (green), respectively, upon vehicle or roscovitine (0.16 μmol·L^−1^) treatment for 6 days (scale bar: 100 µm). **b** Quantification of the percentage of cellular ALP activity in primary murine calvarial osteoblasts upon vehicle or Cdk5 inhibition with roscovitine (*n* = 6). **c** and **d** qPCR analysis of early-stage (*Runx2, Sp7, Alpl*) and late-stage (*Bglap, Ibsp, Spp1*) osteoblast-specific marker gene expression in the primary murine calvarial osteoblasts treated with vehicle or roscovitine for 6 and 14 days, respectively (*n* = 6). **e** Experimental setup for the mice treated with vehicle or roscovitine (150 mg·kg^−1^ three times per week for 14 days). **f** Representative microcomputed tomography (micro-CT) images of whole-, trabecular-, and cortical bone of femurs isolated from the vehicle- or roscovitine-treated mice. Calculated trabecular and cortical parameters of femurs from the vehicle- or roscovitine-treated mice that include **g** percent bone volume - BV/TV (%), **h** trabecular thickness – Tb.Th (µm), **i** trabecular number – Tb.N (per mm), **j** and cross-sectional thickness – Cs.Th (µm) (*n* = 5–6). Bone histomorphometry was performed in femurs from the vehicle- or roscovitine-treated mice, and the following parameters were calculated from trabecular bone: **k** percent osteoblast surface per bone surface - Ob.S/BS (%), **l** and osteoblast number per bone perimeter – Ob.N/B.Pm (per mm). **M** Representative images of tartrate-resistant acid phosphatase (TRAP) staining for osteoclasts (purple) (scale bar: 200 µm), **n** osteoclast surface per bone surface – Oc.S/BS (%) and **o** osteoclast number per bone perimeter – Oc.N/B.Pm (per mm) (*n* = 5). Analysis of bone formation and resorption markers **p** PINP (ng·mL^−1^) and **q** CTX-I (ng·mL^−1^) (*n* = 5–6). Dynamic bone histomorphometry was performed in femurs from the vehicle- or roscovitine-treated mice, and the following parameters were used: **r** representative images of dual calcein labeling (green) (scale bar: 50 µm), **s** mineral apposition rate – MAR/(μm·d^−1^), and **t** bone formation rate – BFR/(µm^3^ per µm^2^ per day). Data are represented as box-and-whisker plots with min. to max. as well as with superimposition of all the data points. Statistical differences between two groups were determined by unpaired homoscedastic two-tailed Student’s *t* test and two-way ANOVA with Sidak’s multiple comparisons test. *P < 0.05, **P < 0.01, ***P < 0.001

Having established that *Cdk5* siRNA knockdown enhances osteoblast differentiation, we investigated the effect of the small molecule Cdk5 inhibitor roscovitine^22^ on osteoblast differentiation. Primary murine calvarial osteoblast treatment with roscovitine enhanced cellular ALP activity (Fig. 3a and b) and significantly increased early- (*Runx2*, *Alpl*) and late-stage (*Bglap*) osteoblast-specific marker gene expression (Fig. 3c and d).

To determine whether roscovitine affects bone mass in vivo, we treated mice with either vehicle or roscovitine for 14 days (Fig. 3e). Microcomputed tomography (micro-CT) demonstrated an increase in the trabecular bone volume (BV/TV), thickness (Tb.Th), and number (Tb.N) and cross-sectional thickness (Cs.Th) in the distal femurs of the roscovitine-treated mice compared with vehicle-treated controls (Fig. 3f–j). Moreover, the treatment had no apparent toxicity, as indicated by the survival and body weight analyses (Fig. S4a and S4b).

Roscovitine treatment induced a marked increase in the number and surface area of osteoblasts in both trabecular and cortical bone (Fig. 3k and l; Fig. S5a and S5b), with no significant differences in osteoclast parameters (Fig. 3m–o; Fig. S5c and S5d). These findings were corroborated by biochemical tests of blood plasma showing increased levels of the bone formation marker N-terminal propeptide of type I procollagen (PINP) without effects on the bone resorption marker C-terminal telopeptides of type I collagen (CTX-I) (Fig. 3p and q). Consistent with these findings, dynamic histomorphometric analysis of the dual calcein-labeled bone sections showed a trend toward an increased mineral apposition rate (MAR) and bone formation rate (BFR) upon roscovitine treatment compared with those of the vehicle-treated controls (Fig. 3r-t).

Furthermore, we investigated the in vitro effect of roscovitine on osteoclastogenesis. Consistent with the in vivo findings, roscovitine did not influence osteoclast numbers (Fig. S6a and S6b). However, the expression of some of the osteoclast-related genes (*Acp5*, *Atp6v0d2*, *car2*, *Dcstamp*) was reduced (Fig. S6c–f). In contrast, the expression of the majority of the osteoclastic genes (*Calcr*, *Clcn7*, *Ctsk*, *Mmp9*, *Nfatc1*, *Ocstamp*, *Ostm1*, *Tnfrsf11a*) remained unchanged upon roscovitine treatment (Fig. S6g-n).

**Fig. 4:**
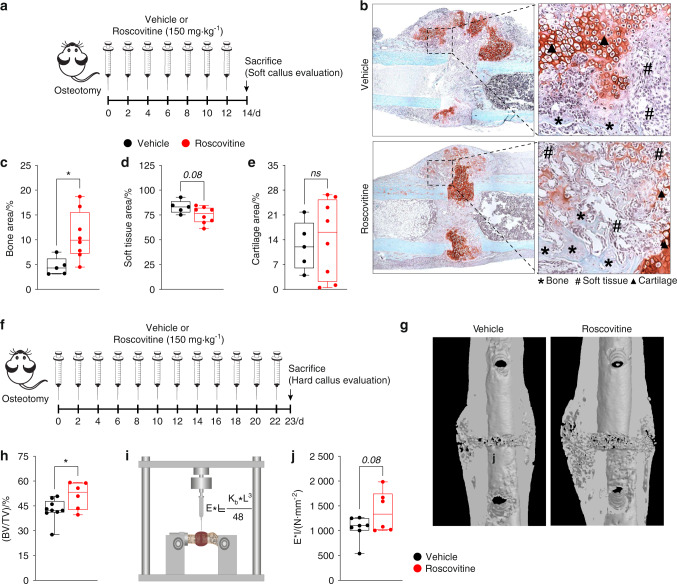
Roscovitine inhibition of Cdk5 improves fracture healing. **a** Experimental setup for the fracture model to evaluate soft calli after the mice were osteotomized followed by treatment with vehicle or roscovitine (150 mg·kg^−1^ every second day for 14 days). **b** Representative images of safranin O/fast green staining of the fracture callus from the mice after 14 days of treatment with vehicle or roscovitine. Quantification of **c** bone area (%), **d** soft tissue area (%), and **e** cartilage area (%) from the mice treated with vehicle or roscovitine (*n* = 5–8). **f** Experimental setup for the fracture model to evaluate hard calli after the mice were osteotomized followed by treatment with vehicle or roscovitine (150 mg·kg^−1^ four times per week for 23 days). **g** Representative microcomputed tomography (micro-CT) images of calli isolated from the mice treated with vehicle or roscovitine for 23 days. **h** Quantification of percent bone volume - BV/TV (%) from calli isolated from the mice treated with vehicle or roscovitine for 23 days. **i** Graphical representation of biomechanical testing (three-point bending test). **j** Bending stiffness (E*I) analysis of calli from the osteotomized mice after treatment with vehicle or roscovitine for 23 days. Data are represented as box-and-whisker plots with min. to max. as well as with superimposition of all the data points. Statistical differences between groups were determined by unpaired homoscedastic two-tailed Student’s *t* test. *P < 0.05, **P < 0.01, ***P < 0.001

Given the profound effect of roscovitine on osteoblastogenesis, we investigated whether it affects bone regeneration during fracture healing. A femur osteotomy was performed, and the mice were treated with either vehicle or roscovitine for 14 or 23 days to evaluate fracture callus formation (Fig. 4a and f). After 14 days, when the mouse fracture callus predominantly consisted of fibrous and cartilage tissues^23^, roscovitine treatment increased the bone fraction, whereas the soft tissue fraction was reduced (Fig. 4b–d). The amount of cartilage was unaltered (Fig. 4b and e). To evaluate the effect of roscovitine on hard callus formation, we extended the treatment to 23 days post-osteotomy (Fig. 4f). The roscovitine-treated mice displayed a significantly increased BV/TV value in the fracture callus, as shown by micro-CT (Fig. 4g and h), thus leading to improved flexural rigidity - a measure of the mechanical properties (Fig. 4i and j).

Collectively, these data demonstrated that roscovitine treatment augments in vitro osteoblast differentiation and increases bone mass via increased osteoblastogenesis, as opposed to impaired osteoclastogenesis. Additionally, roscovitine treatment improved bone formation during fracture healing.

### Cdk5 suppresses osteoblast differentiation through the MAPK pathway

**Fig. 5:**
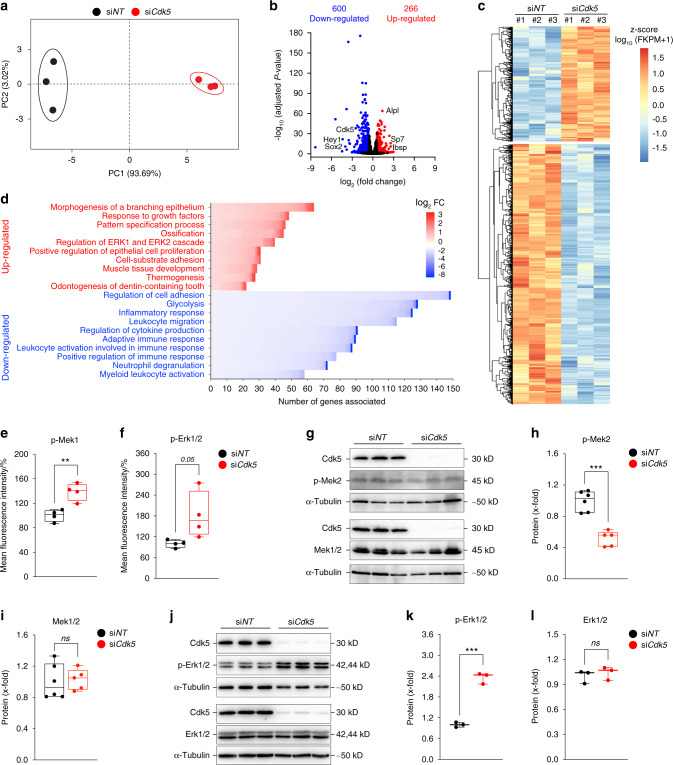
Cdk5 suppresses osteoblast differentiation through inhibition of the Erk pathway. **a** Principal component analysis (PCA) of the RNA-seq data based on the gene transcription of six independent samples. Primary murine calvarial osteoblasts treated with si*NT* or si*Cdk5* for 8 days were distinctly clustered within the first principal component (PC1), explaining 93.69% of the observed variation. **b** Volcano plot: the red dots indicate genes with significantly upregulated expression (266), the blue dots indicate genes with significantly downregulated expression (600), and the black dots indicate non-significant differentially expressed genes (log_2_ FC ≥ 0.58 and ≤ −0.58 and FDR (adjusted *p* value) < 0.05). **c** Hierarchical clustering analysis of all differentially regulated genes from the primary murine calvarial osteoblasts transfected with si*NT* or si*Cdk5* for 8 days. **d** Genes with differentially upregulated and downregulated expression were independently subjected to Metascape analysis. The number of genes in each of the top ten upregulated and downregulated enriched Gene Ontology terms present in our dataset and sorted on the basis of log_2_ FC were plotted. Bio-Plex assays of the primary murine calvarial osteoblasts transfected with si*NT* or si*Cdk5* for 8 days. Percentage fluorescence intensity quantification of **e** p-Mek1 (Ser^217^/Ser^221^) and **f** p-Erk1/2 (Thr^202^/Tyr^204^). **g** Western blots of pMek2 (Thr^394^) and Mek1/2 from the primary murine calvarial osteoblasts treated with si*NT* or si*Cdk5* for 8 days. Protein quantification of **h** p-Mek2 (Thr^394^) and **i**, Mek1/2. **j**, Western blots of pErk1/2 (Thr^202^/Tyr^204^) and Erk1/2 from the primary murine calvarial osteoblasts treated with si*NT* or siCdk5 for 8 days. Protein quantification of **k** p-Erk1/2 (Thr^202^/Tyr^204^) and **l**, Erk1/2. Data are represented as box-and-whisker plots with min. to max. as well as with superimposition of all of the data points. Statistical differences between groups were determined by unpaired homoscedastic two-tailed Student’s *t* test. *P < 0.05, **P < 0.01, ***P < 0.001

To investigate Cdk5 modulation of signaling pathways associated with osteoblast differentiation, we analyzed the transcriptomes of the si*NT*- and si*Cdk5*-treated primary murine calvarial osteoblasts. Subsequently, the osteoblast differentiation-associated signaling pathways affected by *Cdk5* knockdown were investigated by RNA-Seq analysis. Principal component analysis (PCA) demonstrated distinct mRNA expression clustering between the si*NT* control and *Cdk5*-depleted conditions, which were analyzed in triplicate (Fig. 5a, Table S2). We identified 866 differentially expressed genes. A total of 266 genes had upregulated expression and 600 had downregulated expression upon *Cdk5* depletion (Fig. 5b and c; Tables S3 and S4). Metascape analysis revealed biological processes enriched in the genes with downregulated expression that were associated with the regulation of cell adhesion, glycolysis, and the inflammatory response, among others (Table S5). Interestingly, the genes with upregulated expression were mainly associated with morphogenesis of a branching epithelium, response to growth factors, and pattern specification processes, followed by ossification, the last validating our observation of increased osteoblast differentiation (Fig. 5d, Table S6). Regulation of the extracellular signal-regulated kinase 1 (ERK1) and ERK2 cascades was among the top ten biological processes enhanced by si*Cdk5* (Fig. 5d, Table S6). ERK1 and ERK2 are essential regulators of osteoblast differentiation^24^. Upregulated Erk1/2 expression was validated by a Bio-Plex assay of primary murine calvarial osteoblasts transfected with either si*NT* or si*Cdk5*. Increased p-Mek1 (Ser^217^/Ser^221^), p-Erk1/2 (Thr^202^/Tyr^204^), and p-Atf-2 (Thr^71^) phosphorylation and decreased p-Stat3 (Tyr^705^) phosphorylation were observed (Fig. 5e and f; Fig. S7a and S7b). No significant differences were observed in the other investigated signaling molecules, including p-p90rsk (Ser^380^) and p-p38MAPK (Thr^180^/Tyr^182^) (Fig. S7c and S7d). To determine how Cdk5 regulates the Erk pathway, a previous study of adipose tissue showed that the phosphothreonine sites at T394 and T397 on Mek2 were increased by Cdk5^25^. In primary murine calvarial osteoblasts, Cdk5 depletion decreased the inhibitory phosphorylation of Mek2 at T394 (Fig. 5g and h) without affecting the total Mek1/2 protein levels (Fig. 5g and i). This effect eventually enhanced Erk1/2 phosphorylation (Fig. 5j and k) without affecting the total protein level (Fig. 5j and l).

**Fig. 6:**
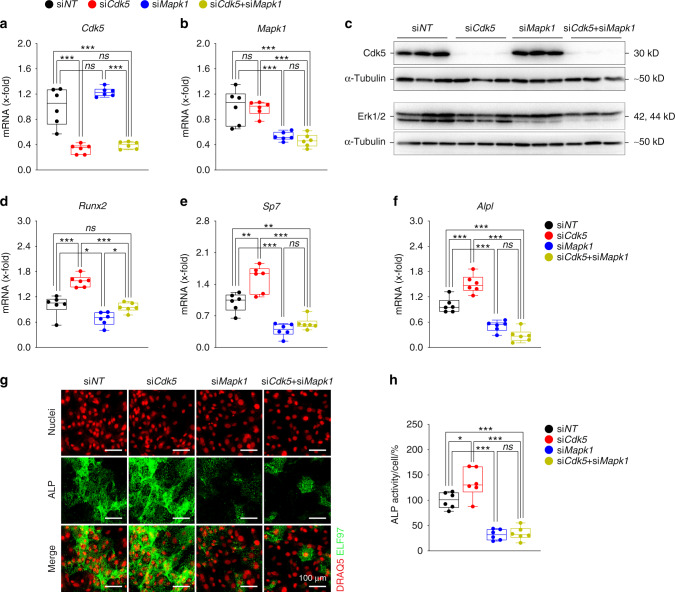
The Erk pathway is essential for Cdk5 regulation of osteoblast differentiation. Primary murine calvarial osteoblasts were transfected with non-targeting siRNA (si*NT*), *Cdk5* siRNA (si*Cdk5*), or *Mapk1* siRNA (si*Mapk1*) or co-transfected with *Cdk5* and *Mapk1* siRNA (si*Cdk5* + si*Mapk1*) for 8 days. qPCR analysis of **a** *Cdk5* and **b** *Mapk1*. Western blots from **c** Cdk5, and Erk1/2. qPCR analysis of **d** *Runx2*, **e** *Sp7*, and **f** *Alpl*. **g** Representative microscopic images showing nuclear and ALP staining with DRAQ5 (red) and ELF97 (green), respectively, of primary calvarial osteoblasts treated with si*NT*, si*Cdk5*, si*Mapk1* or by co-transfection (si*Cdk5* + si*Mapk1*) for 8 days (scale bar: 100 µm). **h** Quantification of the percentage of cellular ALP activity from the primary murine calvarial osteoblasts treated with si*NT*, si*Cdk5*, si*Mapk1*, or si*Cdk5* + si*Mapk1* after 8 days of transfection (*n* = 6). Data are represented as box-and-whisker plots with min. to max. as well as the superimposition of all the data points. Statistical differences between two groups were determined by two-way ANOVA with Sidak’s multiple comparisons test. *P < 0.05, **P < 0.01, ***P < 0.001

To investigate the functional contribution of the Erk pathway to the effects of *Cdk5* depletion, we performed co-transfection of *Cdk5*- and *Mapk1*-specific siRNAs in primary murine calvarial osteoblasts. The co-silencing of *Cdk5* and *Mapk1* successfully reduced Cdk5 and Mapk1 expression at both the mRNA and protein levels (Fig. 6a–c). Notably, the increased expression of early-stage osteoblast-specific markers (*Runx2, Sp7, Alpl*) induced by *Cdk5* knockdown alone was reduced by *Cdk5* and *Mapk1* co-transfection (Fig. 6d–f). Consequently, simultaneous *Cdk5* and *Mapk1* depletion abrogated the increase in ALP activity in primary osteoblasts (Fig. 6g and h). Collectively, these findings demonstrated that Cdk5 depletion increases osteoblastogenesis by enhancing activation of the MAPK/Erk pathway via derepression of Mek activity.

## Discussion

The discovery of novel regulatory targets for small molecule inhibitors that increase bone mass by augmenting bone formation by osteoblasts is extremely important in improving treatment of osteoporosis. Here, an unbiased RNAi screen identified Cdk5, which acts both in vitro and in vivo as a suppressor of osteoblast differentiation. We demonstrated this role by (i) using siRNA in primary murine and human osteoblasts, (ii) Cre-mediated deletion of the *Cdk5* loxP allele in primary murine osteoblasts, and (iii) demonstrating that the inhibition of other Cdk members does not increase osteoblast differentiation. As a proof of concept, we demonstrated that Cdk5 loss or inhibition by the small inhibitor roscovitine enhanced osteoblastogenesis in vitro and in vivo. Furthermore, we demonstrated that Cdk5 mediates these suppressive effects through modulation of the Erk pathway. Therefore, our findings suggest that targeting Cdk5 could serve as a therapeutic approach to treat osteoporosis.

We established here that *Cdk5* is expressed in bone, where it acts as a negative regulator of osteogenesis, a rather unexpected finding, given its well-characterized role in the central nervous system. Cdk5 appears essential in brain development^18^, while in the adult brain, it functions in neuronal survival, cell migration, cortical layering, cytoskeletal dynamics, synaptic plasticity, and axon and dendrite development^26–29^. Cdk5 dysfunction has been implicated in numerous neuronal disorders and neurodegenerative diseases, including Alzheimer’s disease, Parkinson’s disease, amyotrophic lateral sclerosis, prion-related encephalopathies, and Huntington’s disease^30–33^. At the molecular level, Cdk5 mediates its effects on neuronal survival through activation of the PI3K/Akt and MEK/Erk pathways^34,35^. Beyond its roles in the central nervous system, Cdk5 is involved in oncogenesis^36,37^ through its effects on cell proliferation, migration and angiogenesis^38–40^. Cdk5 as a drug target in tumorigenesis is currently under evaluation in phase II clinical trials^41^. Additionally, Cdk5 function is necessary for PPARγ phosphorylation either directly or through inhibition of the MEK/Erk pathway^25^. Recently, this molecule was also shown to affect the activity of other nuclear receptors and their anti-inflammatory action during endotoxemia^42–44^.

Our findings further demonstrated that Cdk5 inhibition both in vitro and in vivo enhanced osteogenesis. Generally, Cdks function in regulating the cell cycle and are essential in diverse processes, including stem-cell self-renewal, transcription, metabolism, and neuronal functions^45,46^. However, in the context of skeletal physiology, the role of Cdks in bone metabolism has not been defined, although Cdk1 was recently shown to be essential in skeletal development^47,48^. A kinome profiling study on osteoblast adhesion to synthetic hydroxyapatite scaffolds indicated a potential role for Cdk5 in osteoblastogenesis^49^. Our data suggest that Cdk5 inhibition at the onset of osteoblastogenesis increases osteoblast differentiation, but this effect is weaker at the later stages. The gradual upregulation of Cdk5 expression during osteoblast differentiation is reminiscent of the expression patterns of other osteoblast differentiation suppressors, including doublecortin-like kinase 1 (Dcamkl1) and kruppel like factor 4 (Klf4), which both show increased expression during osteoblastogenesis^50,51^. Consistent with its inhibitory role, Cdk5 phosphorylation (Cdk5^Tyr15^) decreased during the course of osteoblast differentiation. Therefore, despite the increased total Cdk5 expression during differentiation, the complete loss of phosphorylation indicates an inhibition of Cdk5 function at later stages of differentiation. Strikingly, *CDK5* suppression not only improved differentiation in murine cells but also in osteoblasts grown out of human metatarsal bone, emphasizing the relevance for the human situation.

Furthermore, our data add roscovitine to a group of protein kinase inhibitors, including 603281-31-8, AZD2858, AR79, and AZ13282107 (Gsk-3β inhibitors); SYN1143 and SGX523 (both c-Met receptor tyrosine kinase inhibitors); and SD-208 (Tgf-β type I receptor kinase inhibitor), all of which effectively augment osteoblastogenesis^52–55^.

Although we demonstrated that roscovitine increases bone mass and improves fracture healing in mice, there are potential limitations regarding its use as a Cdk5 inhibitor. Roscovitine, even at low doses, is a potent and selective Cdk5 inhibitor, as exemplified in our in vitro experiments (IC_50_ = 0.16 μmol·L^−1^). Nevertheless, at the higher doses required for in vivo application, roscovitine also inhibits Cdk1 and Cdk2^22,56^. Although a role for Cdk2 in skeletal development has not been reported, osteoblast-specific *Cdk1* deletion was recently shown to impair bone development^47^, which is in contrast to our findings following roscovitine treatment. To exclude any off-target effect of siRNA and roscovitine treatment on osteoblast differentiation, we showed a significant increase in osteoblast differentiation and mineralization in neonatal calvarial osteoblasts from 2- to 5-day-old newborn mice with efficient *Cdk5* deletion using an inducible *Cre-loxP* system. Given the increased bone mass and improved fracture healing after roscovitine treatment, we concluded that roscovitine acts through Cdk5 inhibition. We further demonstrated that roscovitine inhibition of Cdk5 decreased the expression of some of the osteoclast marker genes with no differences in osteoclast numbers. This finding is in contrast to the study of Akiba et al., which described increased osteoclast differentiation of RAW264.7 cells and bone marrow-derived preosteoclasts upon Cdk5 inhibition^57^.

Fracture healing requires both intramembranous and endochondral bone formation to generate a hard callus with good mechanical properties for bridging the bone fragments. Undisturbed osteoblast function is crucial for both of these bone formation processes^58^. Indeed, systemic roscovitine administration increased bone formation at the intermediate healing stage, when bone is generated near the periosteum mainly by intramembranous bone formation but also at the late healing stage, when most of the bone results from cartilage-to-bone transformation^59^. This result is remarkable, considering that, to date, there are few options for systemic treatments to improve bone formation in fracture healing (e.g., teriparatide, a PTH fragment or an antibody neutralizing the anti-osteoanabolic molecule sclerostin)^60,61^.

We further demonstrated that Cdk5 deletion reduced the inhibitory phosphorylation of Mek2 at T394, which eventually enhanced Erk1/2 phosphorylation. These findings are consistent with previous data from brown adipose tissue and primary adipocytes^25^. In addition, we showed that the expression of osteoblast-specific marker genes was enhanced in the *Cdk5*-depleted cells. Intriguingly, earlier studies suggested positive regulation of these markers by the Erk1/2 pathway^62–64^. Together, these data suggest that Cdk5 regulates osteoblast-specific marker gene expression through the Erk1/2 pathway.

In conclusion, we provide evidence for a new function of Cdk5 in skeletal development. The Cdk5 inhibitor roscovitine has shown promising results in several phase I and phase II cancer clinical trials both as a monotherapy and as a combination therapy^41,65,66^. Additionally, roscovitine is currently under evaluation in phase II clinical trials for Cushing’s disease and cystic fibrosis^67^. On the basis of our evidence and the existing extensive clinical trial data, further investigation of roscovitine in bone disease models such as glucocorticoid-induced osteoporosis and menopausal osteoporosis is warranted.

## Materials and methods

### Mice

BALB/cAnNCrl female 11-week-old wild-type mice (Charles River Laboratories, Wilmington, USA) were housed under controlled standard conditions (diurnal lighting conditions with food and water provided ad libitum) in a pathogen-free animal facility at Ulm University. Experimental procedures were approved by the Regierungsprasidium in Tubingen, Germany. For Cdk5 inhibition, mice were injected intraperitoneally (i.p.) with 150 mg·kg^−1^ roscovitine or vehicle (5% dimethyl sulfoxide (DMSO), 10% kolliphor EL, 85% 1x phosphate-buffered saline (PBS)) three times per week for 14 days as previously described^38^. Body weight was measured prior to every roscovitine injection, and the mice were euthanized through intracardial blood withdrawal under deep isoflurane anesthesia at Day 14 to collect the skeletons. *Cdk5*^gtRosaCreERT2^ mice were generated by intercrossing *Cdk5*^flox^ mice^68^ and C57BL/6-GT(ROSA)^26Sortm9(Cre/ESR1)Arte^ mice (Taconic Artemis, Köln, Germany) as previously described^69^.

### Femur osteotomy

The surgeries were performed under general anesthesia with 2% isoflurane (Forene; Abbott Laboratories, Chicago, USA). Male 12-week-old mice received a unilateral femur osteotomy as described previously^23^. Briefly, the right femur was exposed, and the midshaft was osteotomized using a gigli wire saw (0.44 mm). Osteotomy stabilization was achieved using an external fixator (axial stiffness 3.2 N·mm^–1^; RISystem, Davos, Switzerland) that was fitted to the bone using four mini-Schanz screws. Mice received 25 mg·L^–1^ tramadol hydrochloride (Grünenthal, Aachen, Germany) in the drinking water as pain medication from one day pre- until 3 days post-surgery as well as anti-infective treatment with 45 mg·kg^–1^ clindamycin (clindamycin-2-dihydrogenphosphate; Ratiopharm, Ulm, Germany) just prior to surgery. All mice received i.p. injections of either vehicle (PBS/DMSO/Solutol 17:1:2) or 150 mg·kg^–1^ roscovitine (Selleckchem, Houston, USA) solution three to four times weekly for 14 or 23 days. Mice were euthanized under deep isoflurane anesthesia through intracardial blood withdrawal. Fractured right femora were removed for further analysis.

### Microcomputed tomography (micro-CT)

Images of the femurs were acquired by micro-CT using a Bruker Skyscan 1176 (Bruker, Kontich, Belgium) (X-ray voltage = 50 kV, X-ray current = 200 µA, filter = 0.5 mm aluminum, resolution = 9 µm, rotation step = 1°). For reconstruction of trabecular and cortical femurs, the region commenced ~0.215 mm and 1.935 mm from the growth plate in the direction of the metaphysis and was extended by 1.29 mm and 0.43 mm, respectively. Structural indices were determined by Bruker’s Skyscan CT Analyzer (CTan) software. The calculated trabecular and cortical parameters are presented in Fig. 3. Three-dimensional models were created using CT-volume.

In fractured femurs, the region of interest was defined as the periosteal callus between both inner pin holes. The BV/TV was determined using a global threshold of 642 mgHA·cm^–2^ as described previously^70^.

### Biomechanical testing of the fracture callus

Directly after euthanasia, both femurs underwent biomechanical testing using a nondestructive three-point bending test in a universal material testing machine (Z10, Zwick Roell, Ulm, Germany) as described previously^23,71^. Briefly, the proximal end of the femur was fixed in an aluminum cup, which in turn was fixed to a hinge joint of the three-point bending setup in the material testing machine (Fig. 4i). The femur condyles rested unfixed on a bending support. The bending load was applied to the middle of the callus up to a maximum load of 2 N. Flexural rigidity (EI) was calculated from the slope (*k*) of the linear region of the force–displacement curve.

### Bone histomorphometry

Femurs were isolated, fixed in 4% p-formaldehyde (PFA) for 3 days, decalcified with 15% EDTA for 10 days, paraffin-embedded, and sectioned (7 µm). Prior to tartrate resistant acid phosphatase (TRAP) staining, the femur sections were deparaffinized, incubated in TRAP-staining solution for 1 h, washed, counterstained with hematoxylin for 3 min, and mounted with Aquatex aqueous mounting media (Santa Cruz, Houston, USA). Static histomorphometry was performed on TRAP-stained sections for osteoblasts and osteoclasts and analyzed using the OsteoMeasure high-resolution digital video system (OsteoMetrics, Inc., Decatur, USA).

For dynamic histomorphometry, mice were injected (i.p.) with calcein solution, as described previously^72,73^, at 9 and 2 days prior to skeleton collection. Femurs were isolated, stripped of muscle and other soft tissues, fixed in 4% PFA, and embedded in methacrylate as previously described^74^. Histomorphometric analysis of 7 µm sections was performed according to standard procedures using the Osteomeasure system. The following parameters were measured: MAR and BFR. The results are shown in Fig. 3.

### Enzyme-linked immunosorbent assays (ELISAs)

Blood was collected in heparin-coated tubes, incubated at room temperature (RT) for 15 min, and centrifuged at 2 000 × *g* for ten min at RT to collect the supernatant (plasma). PINP and CTX-I ELISAs (Immunodiagnostic Systems, Boldon, UK) were performed according to the manufacturer’s instructions.

### Osteoblast differentiation

Primary calvarial osteoblasts were collected from mouse calvaria of 2- to 5-day-old pups, as previously described^11^. Briefly, following isolation, the calvariae were placed in 1 mL of digestion solution (0.2% w/v of both Collagenase A (Roche, Basel, Switzerland) and Dispase II (Roche, Basel, Switzerland)) and incubated at 37 °C with shaking at 700 r·min^−1^ for 10 min. The first supernatant (fraction 1) was discarded. The digestion was repeated four further times (fractions 2–5), and the supernatant of the combined fractions was collected in 15 mL Falcon tubes containing 500 µL of fetal bovine serum (FBS) (GE Healthcare, Chicago, USA). The collected supernatant was centrifuged at 1 500 r·min^−1^ for 5 min at RT. The cell suspension from each pup was resuspended in 3 mL of complete medium [α-MEM (Thermo Fisher Scientific, Waltham, USA) with 10% FBS and 1% penicillin/streptomycin (Sigma-Aldrich, St. Louis, USA)] and was plated in a six-well plate using one well per calvaria. The cells were maintained in an incubator under 5% CO_2_ at 37 °C overnight. The following day, any nonadherent cells were removed by replacing the cell culture medium with fresh complete medium. The cells were allowed to grow for 2–3 days until reaching ~80% confluency.

For all experiments, primary murine calvarial osteoblasts were seeded at a confluency of 12 000 cells per cm^2^, and 48 h after seeding, the medium was replaced by osteogenic induction medium [100 μg·mL^−1^ (+)-sodium L-ascorbate and 5 mmol·L^−1^ β-glycerophosphate (both Sigma-Aldrich, St. Louis, USA)]. Treatment with roscovitine (0.16 μmol·L^−1^) was performed in osteogenic induction medium. Ethanol vehicle at 0.01% was used as a control. Roscovitine treatment was performed every third day until the termination of the experiment.

Primary osteoblasts isolated by sequential digestions from the calvaria of neonatal *Cdk5*^flox^ and *Cdk5*^gtRosaCreERT2^ pups were cultivated as described above. The cells were exposed to 1 μmol·L^−1^ 4-hydroxytamoxifen (4-OHT) on the second day following isolation for 3 days. Subsequently, the cells were trypsinized, seeded, and allowed to differentiate by adding osteogenic induction medium as described above.

### Human primary cells

The bone samples used were resected from the medial head of the first metatarsal bone during operative foot alignment correction by Chevron osteotomy. Regarding the surgical gain of samples, bone resection as described above is essential for Chevron osteotomy, such that no further damage or extra operation time is needed. Ethical approval for this study was obtained from the ethical commission of Ulm University (306/19). The bone sample was cut into ~2 mm Ø pieces, transferred into a 50-mL falcon, and washed several times vigorously with 1× PBS until it appeared white. Subsequently, the bone pieces were transferred into 10-cm petri dishes (10–12 pieces per dish) containing 7 mL of complete DMEM [DMEM (Thermo Fisher Scientific, Waltham, USA) with 10% FBS and 1% penicillin/streptomycin]. The cells were incubated at 37 °C under 5% CO_2_ (note: it can take up to 10 days before the first cells appear). Ten milliliters of complete DMEM was exchanged every third day for 6–8 weeks. Subsequently, the bone pieces were washed out from the dishes by using 1× PBS, and the attached cells were trypsinized and reseeded in new 10-cm dishes. The cells were cultured again for ~2 weeks until they reached 80% confluency.

For the siRNA experiment, human primary osteoblasts were reverse transfected and seeded at a confluency of 12 000 cells per cm^2^. The final siRNA concentration and percent RNAiMAX (Life Technologies, Carlsbad, USA) used were 20 nmol·L^−1^ and 0.125%, respectively. After 48 h, the medium was replaced with osteogenic induction medium, which was exchanged every third day until the termination of the experiment.

### siRNA transfection

SMARTpool siRNA targeting mouse and human non-targeting #2 (Cat. D-001206-13-05), Cdk5 (Cat. M-040544-01-0005), Mapk1 (Cat. M-040613-01-0005), and CDK5 (Cat. M-003239-01-0005) were purchased from Dharmacon (Thermo Fisher Scientific, Waltham, USA). siRNA transfection was performed at a final siRNA concentration of 20 nmol·L^−1^ and 0.125% RNAiMAX as previously described^11^. The siRNA sequences used are given in Table S7.

### High-content RNAi screening of a kinase library

The SMARTpool siRNA kinase library was purchased from Dharmacon. Reverse transfection was performed using the Tecan Freedom EVO pipetting workstation (Tecan Life Sciences, Maennedorf, Switzerland). This process was followed by the addition of a cell suspension using a BioTek cell dispenser (LabX, Midland, Canada). The cells were cultured for 8 days, followed by fixation, staining, image acquisition and analysis, data analysis, and finally hit selection as previously described^11^.

### Quantitative ALP staining

For quantitative ALP, primary murine calvarial osteoblasts were seeded in 384-well plates and differentiated by adding osteogenic induction medium. The cells were fixed, stained, and analyzed as previously described^11^. For in vitro Cdk5 inhibition, primary murine calvarial osteoblasts were treated with either vehicle or roscovitine (0.16 μmol·L^−1^) for 6 and 14 days.

### PrestoBlue, ALP, and Alizarin Red S staining

Cell viability, ALP staining, and Alizarin Red S staining were determined using PrestoBlue cell viability reagent (Life Technologies, Carlsbad, USA), ALP kit (Sigma-Aldrich, St. Louis, USA), and 1% Alizarin Red S (Sigma-Aldrich, St. Louis, USA) according to the manufacturers’ instructions and as previously described^11^.

### Osteoclast differentiation

Bone marrow cells were isolated from femurs and tibias of 13-week-old male mice, and 2.5 × 10^5^ and 2.5 × 10^6^ cells were seeded in a 24-well plate and 6-cm dishes, respectively. Osteoclast differentiation was performed in α-MEM containing 10% FBS and 1% penicillin/streptomycin and supplemented with 50 ng·mL^−1^ RANKL (R&D Systems, Minneapolis, USA) and 20 ng·mL^−1^ M-CSF (R&D Systems, Minneapolis, USA) for 8 days, and the media was changed every second day. Roscovitine (0.16 μmol·L^−1^) treatment was performed every second day until the termination of the experiment. TRAP staining was performed using a TRAP kit (Sigma-Aldrich, St. Louis, USA), with osteoclasts defined as TRAP-positive cells with ≥3 nuclei. Osteoclast numbers were quantified using Osteomeasure software.

### Bio-Plex assay

Protein lysates (10 µg per sample) were prepared from primary murine calvarial osteoblasts after 8 days of transfection with non-targeting siRNA or *Cdk5*-specific siRNA using a Bio-Plex cell lysis kit (Bio-Rad Laboratories, Hercules, USA). The phosphorylation levels of the proteins listed in Fig. 5e and f and Fig. S7a–d were determined using the Bio-Plex Pro Cell Signaling panel (Bio-Rad Laboratories, Hercules, USA) according to the manufacturer’s instructions. The data were analyzed using Bio-Plex Manager 6.1 software (Bio-Rad Laboratories, Hercules, USA).

### RNA extraction, cDNA synthesis, and quantitative real-time polymerase chain reaction (PCR)

Total RNA was extracted from organs using TRIzol reagent (Life Technologies, Carlsbad, USA) and from cells using the RNeasy kit (Qiagen, Hilden, Germany) according to the manufacturers’ instructions. Reverse transcription of 1 000 ng RNA from both cells and organs was performed using the RevertAid H Minus reverse transcriptase kit (Fermentas, Waltham, USA). qPCR was performed on a ViiA 7 system (Applied Biosystems, Waltham, USA), and the relative mRNA concentrations normalized to that of β-actin were calculated by the 2^−ΔΔCt^ method. The primer sequences used in qPCR are shown in Table S8.

### RNA-seq and bioinformatics analysis

Following RNA isolation using the RNeasy kit (Qiagen, Hilden, Germany), the RNA concentration and integrity were determined using a 2100 Bioanalyzer and the RNA 6000 Nano kit (Agilent Technologies, Santa Clara, USA). RNA samples with an A_260_/A_280_ ratio of 1.9–2.0 and an RNA integrity number (RIN) > 9.5 were used. RNA sequencing by Illumina (HiSeq 2500, Illumina, San Diego, USA) was performed by Novogene (Novogene Company Limited, Cambridge, UK). The original image data file from high-throughput sequencing was transformed to sequenced reads by CASAVA base recognition. Sequence adapters and low-quality reads were removed using fastp. Quality control analysis of raw sequence data was performed with fastp. Subsequently, sequencing reads were mapped to the mouse reference genome (ensembl_mus_musculus_grcm38_p6_gca_000001635_8) using the STAR program. The expression quantity of each transcript was calculated by analysis of the alignment results using the feature counts, and the alignment results were analyzed by STAR. The FKPM method was applied to quantify gene expression. PCA was performed to identify the variability and repeatability of samples in R using RStudio with pcaExplorer^75^. The overall distribution of differentially regulated genes was visualized using a volcano plot [log_2_ FC ≥ 0.58 and ≤ -0.58 and FDR (adjusted *P* value) <0.05] in GraphPad Prism (v9.0.0). Hierarchical clustering analysis of all differentially regulated genes from primary murine calvarial osteoblasts treated with nontargeting siRNA or *Cdk5*-specific siRNA for eight days was performed in R using RStudio with pheatmap (RRID:SCR_016418). Gene Ontology (GO) analysis of genes with differentially up- and downregulated expression was performed using Metascape^76^. The top clusters from the GO terms from both the up- and downregulated groups present in our dataset were sorted and plotted on the basis of log_2_ FC using RStudio.

### Protein isolation, quantification, and western blotting

Total cellular protein was extracted using RIPA lysis buffer, quantified using the Pierce BCA protein assay kit (Thermo Fisher Scientific, Waltham, USA), and subjected to western blotting as previously described^11^ using antibodies against Cdk5, pCdk5, Runx2, Mek1/2, pErk1/2, Erk1/2 (all from Cell Signaling Technology, Danvers, USA), Sp7 (Abcam, Cambridge, UK), pMek2 (Life Technologies, Carlsbad, USA), and α-tubulin (Sigma-Aldrich, St. Louis, USA). The band intensity of western blots was quantified using Fiji ImageJ.

### Statistical analysis

Data are represented as box-and-whisker plots with min. to max. as well as superimposition of all the data points. Statistical differences between two groups were determined by unpaired homoscedastic two-tailed Student’s *t* test and two-way ANOVA with Sidak’s multiple comparisons test. A *p* value less than 0.05 was considered to be statistically significantly, *P < 0.05, **P < 0.01, ***P < 0.001.

## Supplementary information

Supplemental Figures

Supplementary Table 1

Supplementary Table 2

Supplementary Table 3

Supplementary Table 4

Supplementary Table 5

Supplementary Table 6

Supplementary Table 7

Supplementary Table 8

## Data Availability

The datasets used and/or analyzed during the current study are available from the corresponding author on reasonable request.
