# Supplementary material for: Inhibition of Cdk5 increases osteoblast differentiation and bone mass and improves fracture healing
Source: Bone Res. 2022 Apr 6;10:33. doi: 10.1038/s41413-022-00195-z (PMC8983726; doi:10.1038/s41413-022-00195-z)
Supplement: Supplementary file 1 — Supplemental Figures [file 41413_2022_195_MOESM1_ESM.docx]

**Inhibition of Cdk5 increases osteoblast differentiation, bone mass and improves fracture healing**

*Running Head: Inhibition of Cdk5 regulates bone mass*

Mubashir Ahmad^1^, Benjamin Thilo Krüger^2^, Torsten Kroll^3^, Sabine Vettorazzi^1^, Ann-Kristin Picke^1^, Florian Mengele^4^, Sooyeon Lee^1^, Sayantan Nandi^1^, Dilay Yilmaz^1^, Miriam Stolz^1^, Naveen Kumar Tangudu^1,5^, David Carro Vázquez^1,6^, Johanna Pachmayr^7^, Ion Cristian Cirstea^1^, Maja Vujic Spasic^1^, Aspasia Ploubidou^3^, Anita Ignatius^2^, Jan Tuckermann^1,8,*^

^1^ Institute of Comparative Molecular Endocrinology (CME), Ulm University, Helmholtzstrasse 8/1, 89081 Ulm, Germany.

^2^ Institute of Orthopaedic Research and Biomechanics, Ulm University, Helmholtzstrasse 14, 89081 Ulm, Germany.

^3^ Leibniz Institute on Aging – Fritz Lipmann Institute (FLI), Beutenbergstrasse 11, D-07745 Jena, Germany.

^4^ Praxisklinik fur Orthopädie, Unfall- und Neurochirugie Prof. Bischoff/Dr. Spies/Dr. Mengele, 89231 Neu-Ulm, Germany.

^5^ UPMC Hillman Cancer Center, Department of Pharmacology and Chemical Biology, University of Pittsburgh, 15232 PA, USA.

^6^ Present address: TAmiRNA GmbH, Leberstrasse 20, 1110 Vienna, Austria.

^7^ Paracelsus Medizinische Privatuniverstät, Institute of Pharmacy, Strubergasse 21, 5020 Salzburg, Austria.

^8^ Department of Endocrinology, Ludwig Maximilians University Munich, Munich, 80336, Germany.

**Author for Correspondence:** Prof. Dr. Jan Tuckermann; Institute of Comparative Molecular Endocrinology (CME), Ulm University, Helmholtzstrasse 8/1, 89081 Ulm, Germany; Tel.: +49 (0)731/50-32600, Fax: +49 (0)731/50-32609. Email: [jan.tuckermann@uni-ulm.de](mailto:jan.tuckermann@uni-ulm.de).

**Author contributions**

M.A., B.K., T.K., A.P., A.I., and J.T designed the experiments; M.A., B.K., T.K., S.V., A-K.P., F.M., S.L., S.N., D.Y., M.S., N.K.T., D.C.V., I.C.C., and M.V.S. performed experiments; M.A., B.K., T.K., A.P., A.I., and J.T. performed interpretation of data and statistical analysis; M.A., and J.T. wrote the manuscript. All authors reviewed and approved the final manuscript.

# Supplementary Information


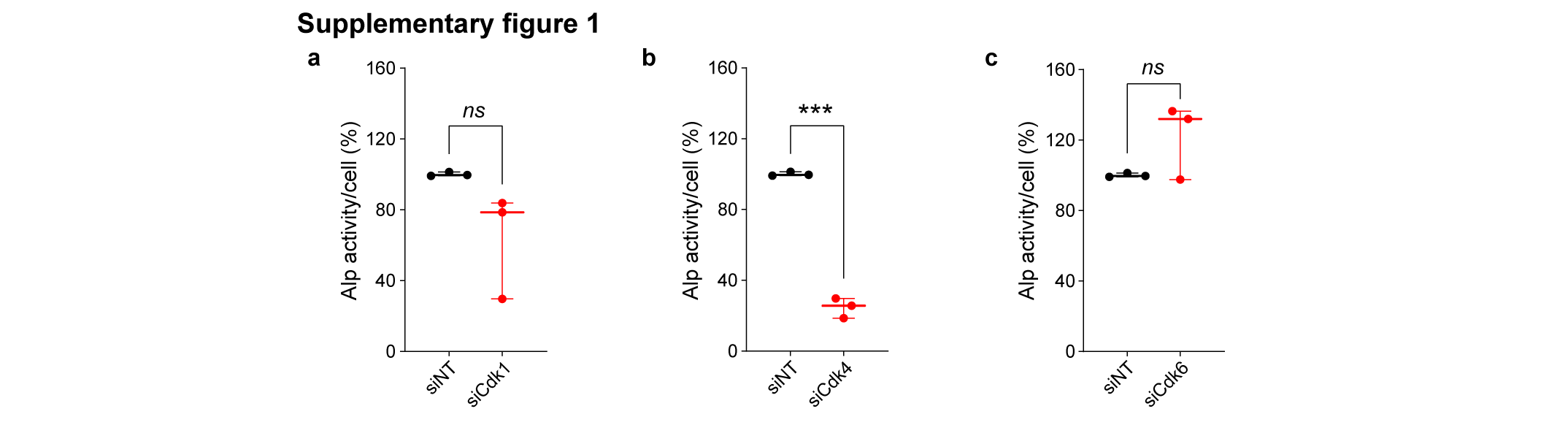


**Supplementary Fig. 1** Cellular alkaline phosphatase activity (ALP) upon siRNA knockdown of *Cdks*. Quantification of percent cellular ALP activity upon siRNA knockdown of: **a**, *Cdk1* **b**, *Cdk4* **c**, *Cdk6*.


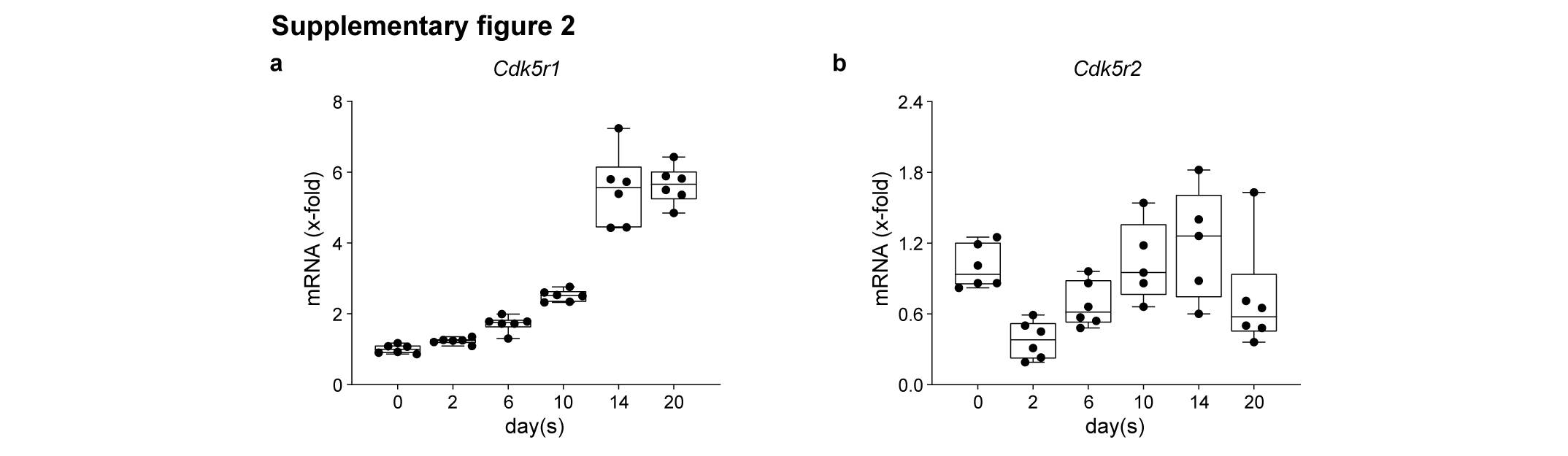


**Supplementary Fig. 2** Expression of *Cdk5* activators in primary murine calvarial osteoblasts. mRNA expression of: **a**, *Cdk5r1 (p35)*, and **b**, *Cdk5r2 (p39)* during the course of osteoblast differentiation in primary murine calvarial osteoblasts (n=5-6).


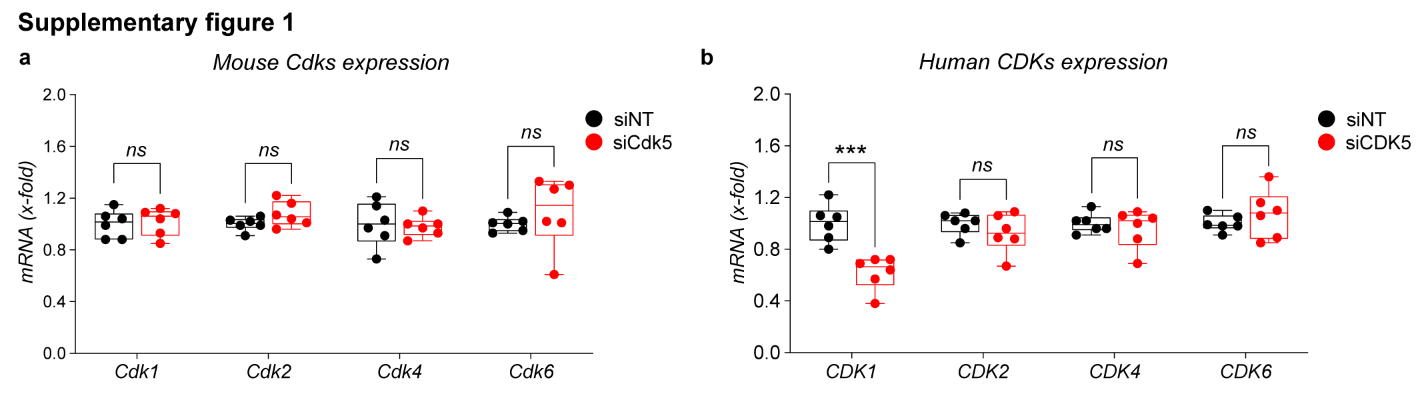


**Supplementary Fig. 3** *Cdks* expression upon silencing of *Cdk5* in primary murine and human osteoblasts. **a**, mRNA expression of *Cdk1*, *Cdk2*, *Cdk4* and *Cdk6* after 8-days of *Cdk5* siRNA transfection quantified by qPCR in primary murine calvarial osteoblasts (n=6). **b**, mRNA expression of *CDK1*, *CDK2*, *CDK4* and *CDK6* after 12-days of *CDK5* siRNA transfection quantified by qPCR in human primary osteoblasts (n=6). Data are represented as box and whisker plots with min to max as well as superimposing all of the data points. Statistical differences between two groups were determined by two-way ANOVA sidak’s multiple comparisons test. **p* < 0.05, ***p* < 0.01, ****p* < 0.001.


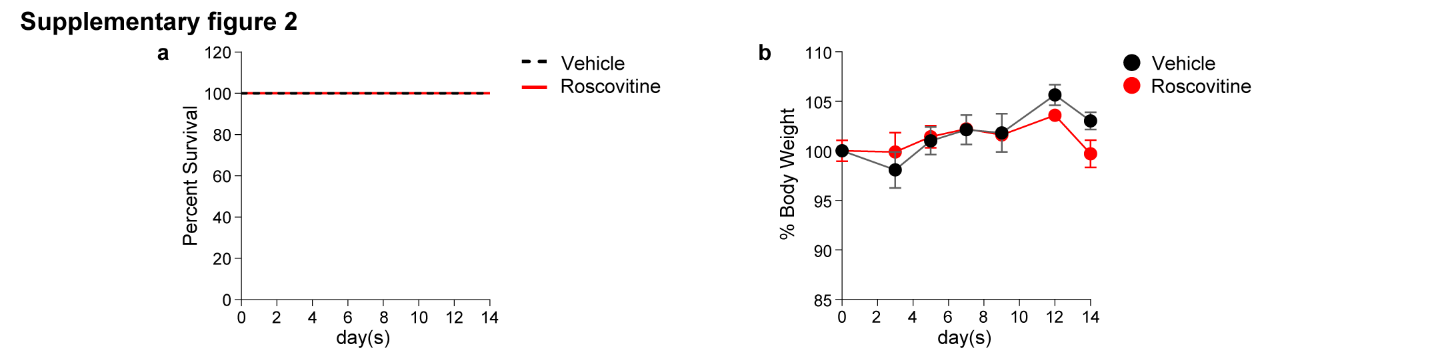


**Supplementary Fig. 4** Cdk5 inhibition with roscovitine has no effect on survivability and body weight. **a,** Percent survivability, and **b**, Body weight from mice treated with vehicle or roscovitine (150 mg/kg three times per week for two weeks) (n=5-6). Statistical differences between groups were determined by unpaired homoscedastic two-tailed student’s *t*-test. **p* < 0.05, ***p* < 0.01, ****p* < 0.001.

^
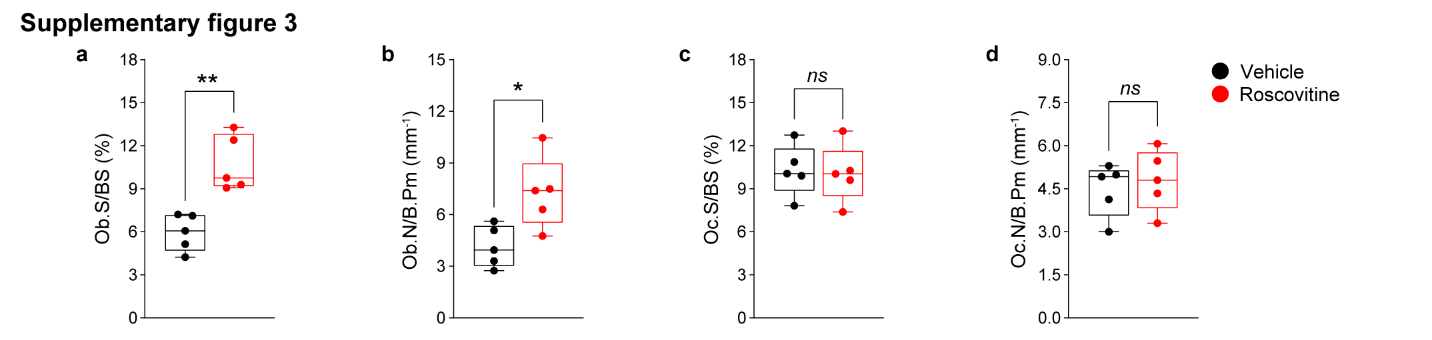
^

**Supplementary Fig. 5** Bone histomorphometry of femoral cortical bone isolated from vehicle- or roscovitine-treated mice. Bone histomorphometry was performed in femurs from vehicle- or roscovitine-treated (150 mg/kg three times per week for two weeks) mice and following parameters were calculated from cortical bone: **a**, percent osteoblast surface per bone surface - Ob.S/BS (%), **b**, osteoblast number per bone perimeter – Ob.N/B.Pm (mm^-1^), **c**, osteoclast surface per bone surface - Oc.S/BS (%), and **d**, osteoclast number per bone perimeter – Oc.N/B.Pm (mm^-1^) (n=5). Statistical differences between groups were determined by unpaired homoscedastic two-tailed student’s *t*-test. **p* < 0.05, ***p* < 0.01, ****p* < 0.001.


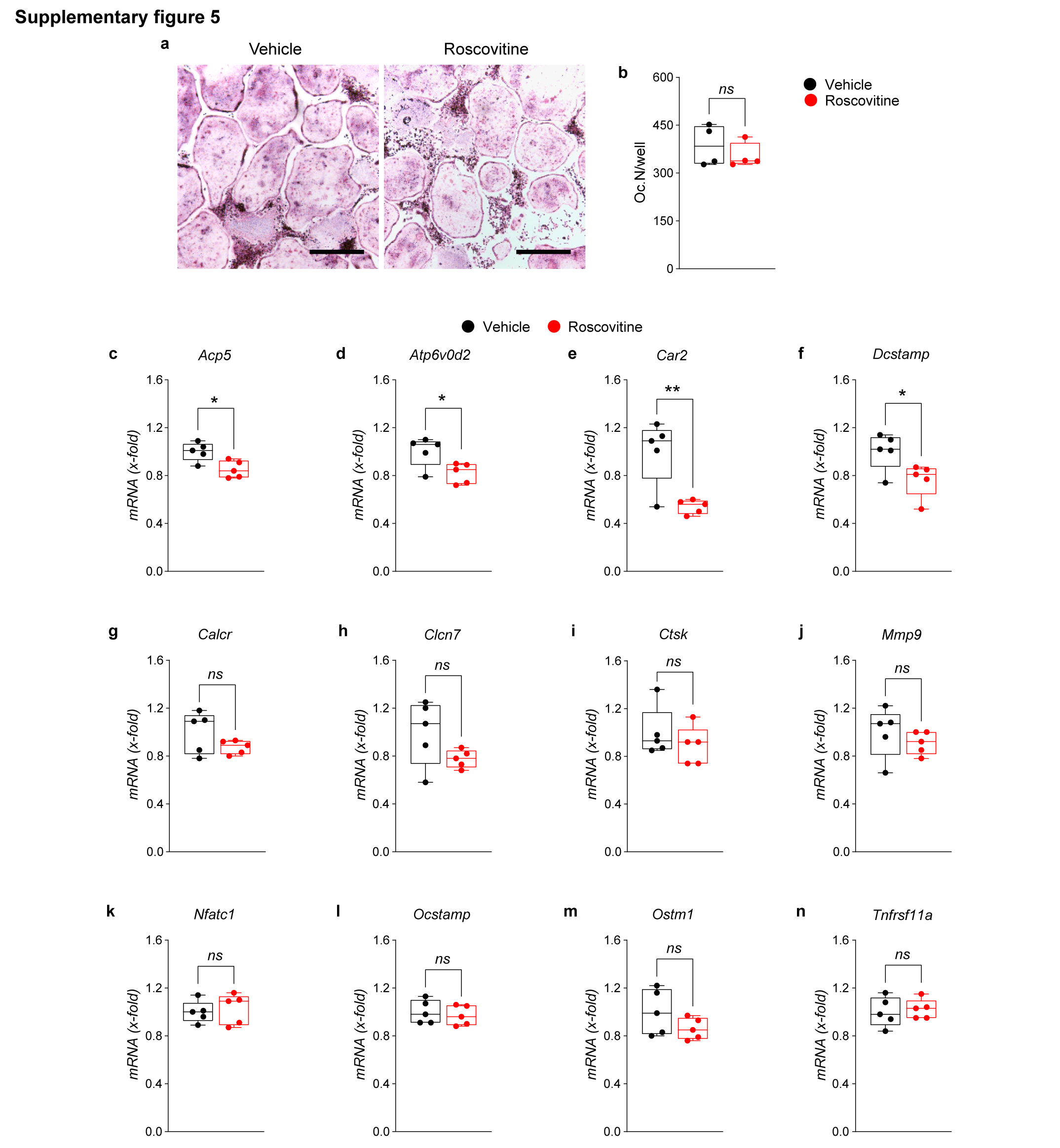


**Supplementary Fig. 6** Impact of Cdk5 inhibition with roscovitine on osteoclastogenesis. **a** and **b**, The representative images and quantitative measurement of TRAP-positive osteoclast numbers per well (Oc.N/well), differentiated from bone marrow cells (BMCs) upon treatment with vehicle or roscovitine (0.16 µM) for 8-days. qPCR analysis of: **c**, *Acp5* **d**, *Atp6v0d2* **e**, *Car2* **f**, *Dcstamp* **g**, *Calcr* **h**, *Clcn7* **i**, *Ctsk* **j**, *Mmp9* **k**, *Nfatc1* **l**, *Ocstamp* **m**, *Ostm* and **n**, *Tnfrsf11a* osteoclasts differentiating from BMCs upon treatment with vehicle or roscovitine (0.16 µM) for 8-days.­


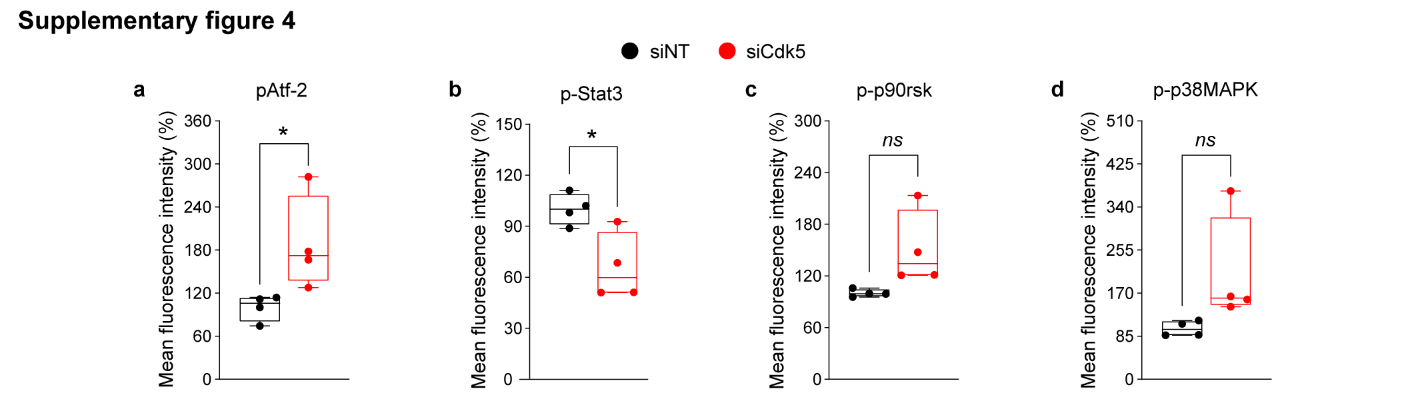


**Supplementary Fig. 7** Bio-Plex Pro^TM^ cell signaling MAPK panel 9-Plex assay. Bio-Plex assay from primary murine calvarial osteoblasts treated with non-targeting siRNA (siNT) or siRNA specific for *Cdk5* (siCdk5) after eight-days of transfection. Percentage fluorescence intensity quantification of: **a**, p-Atf-2 (Thr^71^), **b**, p-Stat3 (Tyr^705^), **c**, p-p90rsk (Ser^380^), and **d**, p-p38MAPK (Thr^180^/Tyr^182^). Data are represented as box and whisker plots with min to max as well as superimposing all of the data points. Statistical differences between groups were determined by unpaired homoscedastic two-tailed student’s *t*-test. **p* < 0.05, ***p* < 0.01, ****p* < 0.001.

**Supplementary Table 1:** List of identified potential 26 suppressors and 156 activators of osteoblast differentiation.

**Supplementary Table 2:** RNA-seq analysis data of primary murine calvarial osteoblasts transfected with either si*NT* or si*Cdk5*.

**Supplementary Table 3:** List of 266 upregulated genes identified by RNA-seq upon *Cdk5* siRNA knockdown in primary murine calvarial osteoblasts.

**Supplementary Table 4:** List of 600 downregulated genes identified by RNA-seq upon *Cdk5* siRNA knockdown in primary murine calvarial osteoblasts.

**Supplementary Table 5:** Metascape analysis showing the list of biological processes enriched by downregulated genes.

**Supplementary Table 6:** Metascape analysis showing the list of biological processes enriched by upregulated genes.

**Supplementary Table 7:** Mouse and human siRNA sequences used in this study.

**Supplementary Table 8:** Oligonucleotide primer sequences from mouse and humans used in real-time polymerase chain reaction.
